# Treatment Choice and Cardiovascular Risk in Chronic Spontaneous Urticaria

**DOI:** 10.1002/clt2.70219

**Published:** 2026-10-21

**Authors:** Ralf J. Ludwig, Diamant Thaçi, Philip Curman

**Affiliations:** ^1^ Lübeck Institute of Experimental Dermatology University of Lübeck Lübeck Germany; ^2^ Department of Dermatology University‐Hospital Schleswig‐Holstein (UKSH) Lübeck Germany; ^3^ Institute and Comprehensive Center for Inflammation Medicine University of Lübeck Lübeck Germany; ^4^ Dermato‐Venereology Clinic Karolinska University Hospital Stockholm Sweden; ^5^ Dermatology and Venereology Division Department of Medicine (Solna) Karolinska Institutet Stockholm Sweden; ^6^ Department of Medical Epidemiology and Biostatistics Karolinska Institutet Stockholm Sweden

To the Editor,

Chronic spontaneous urticaria (CSU) is increasingly recognized as a systemic inflammatory disease [1], including elevated risks for cardiovascular diseases (CVD) compared with the general population, with adjusted hazard ratios of 1.25–1.86 across six cardiovascular outcomes, including major adverse cardiovascular events [2, 3], yet the impact of treatment choice on CVD has not been addressed in CSU. Given the central role of inflammation in CSU pathobiology and cardiovascular disease (CVD), it is biologically plausible that effective disease control could mitigate CVD risk.

To address this, we used the US Collaborative Network of TriNetX, and performed a retrospective matched cohort study [4] comparing CSU treated with second‐generation antihistamines (sgAH), omalizumab (OMA), or oral corticosteroids (oCS). Three pairwise matched analyses were conducted: OMA versus sgAH, OMA versus oCS, and sgAH versus oCS. Adults were included if they had ≥ 2 urticaria codes ≥ 6 weeks apart and received the treatment of interest in conjunction with an urticaria diagnosis. Two treatments (OMA, oCS) were reciprocally excluded from index up to 3 years thereafter. Second‐generation antihistamines were not treated as an exclusion criterion, reflecting their guideline‐recommended continuation alongside omalizumab; comparisons therefore contrast treatment strategies as applied in practice rather than mutually exclusive exposures. For each comparison, treatment cohorts were matched 1:1 using propensity‐score covariates (Supporting Information S1: Table S1) and identical follow‐up windows were applied to ensure comparability. Three predefined analyses were performed: primary analysis (1 day–3 years), sensitivity analysis excluding the first 3 months (S1), and a mandatory follow‐up requiring a healthcare visit between 1 and 3 years after index (S2) (Supporting Information S1: Figure S1).

Across comparisons, baseline characteristics were well‐balanced after matching with cohort sizes of ∼3300 for OMA groups and ∼31,000 for sgAH/oCS (Table 1; Supporting Information S1: Table S1). The primary outcome was any CVD, defined as a composite of major cardiovascular diagnoses assessed using time‐to‐event Cox models. Secondary outcomes included major adverse cardiovascular events (MACE), venous thromboembolism (VTE), and other CVD (Supporting Information S1: Table S2). Any CVD showed a clear pattern across all analyses: OMA was consistently associated with lower CVD risk compared with both oCS and sgAH. Compared with oCS, OMA users had substantially lower absolute risk across all analysis windows (e.g., primary: 3.55% vs 8.06%; S1: 2.75% vs 6.81%; S2: 4.21% vs 9.22%), with adjusted hazard ratios (aHRs) ranging 0.51–0.54 (all *p* < 0.001) (Figure 1). Lower risk versus sgAH followed the same trend. Furthermore, sgAH demonstrated modestly lower CVD risk compared to oCS. These findings were directionally consistent across all predefined time windows.

**TABLE 1:** Summarized baseline characteristics.

Omalizumab versus oral corticosteroids						
Characteristic	Before matching			After matching		
	OMA	oCS	Std. Diff.	OMA	oCS	Std. Diff.
*n*	3303	44,614	—	3302	3302	—
Age at index (years, SD)	43.2 ± 16.0	44.1 ± 15.9	0.051	43.2 ± 16.0	43.6 ± 16.0	0.021
Female (%)	76.5	76.9	0.009	76.6	77.4	0.021
Asian (%)	4.1	8.4	0.175	4.1	4.2	0.003
Black (%)	9.4	16.1	0.201	9.4	8.5	0.034
Hispanic or Latino (%)	7.5	9.6	0.075	7.5	6.6	0.035
White (%)	73.3	63.9	0.203	73.3	75.1	0.042

Omalizumab versus second‐generation antihistamines						
Characteristic	Before matching			After matching		
	OMA	sgAH	Std. Diff.	OMA	sgAH	Std. Diff.
*n*	3303	33,099	—	3293	3293	—
Age at index (years, SD)	43.2 ± 16.0	42.7 ± 15.9	0.035	43.2 ± 16.0	44.0 ± 16.3	0.046
Female (%)	76.5	77.1	0.013	76.5	77.5	0.024
Asian (%)	4.1	10.3	0.239	4.2	3.8	0.020
Black (%)	9.4	18.9	0.273	9.5	8.7	0.027
Hispanic or Latino (%)	7.5	9.5	0.070	7.5	7.4	0.006
White (%)	73.3	54.9	0.390	73.2	74.3	0.026

Second‐generation antihistamines versus oral corticosteroids						
Characteristic	Before matching			After matching		
	sgAH	oCS	Std. Diff.	sgAH	oCS	Std. Diff.
*n*	33,099	44,614	—	31,602	31,602	—
Age at index (years, SD)	42.7 ± 15.9	44.1 ± 15.9	0.087	42.9 ± 15.9	42.6 ± 15.7	0.014
Female (%)	77.1	76.9	0.004	77.1	77.1	< 0.001
Asian (%)	10.3	8.4	0.067	10.4	10.0	0.015
Black (%)	18.9	16.1	0.073	19.1	18.3	0.020
Hispanic or Latino (%)	9.5	9.6	0.005	9.6	9.6	0.002
White (%)	54.9	63.9	0.184	56.4	57.5	0.022

*Note:* Variables are reported before and after matching for the primary study cohorts. Definitions for characteristics are in International classification of diseases, 10th edition, clinical modification (ICD‐10‐CM) codes for comorbidities and RxNorm for drugs. Complete baseline characteristics including all variables used for propensity‐score matching are found in Supporting Information S1: Table S1.

Abbreviations: oCS: oral corticosteroids; OMA: omalizumab; SD: standard deviation; sgAH: second‐generation antihistamines; Std. Diff.; standardized difference.

**FIGURE 1:**
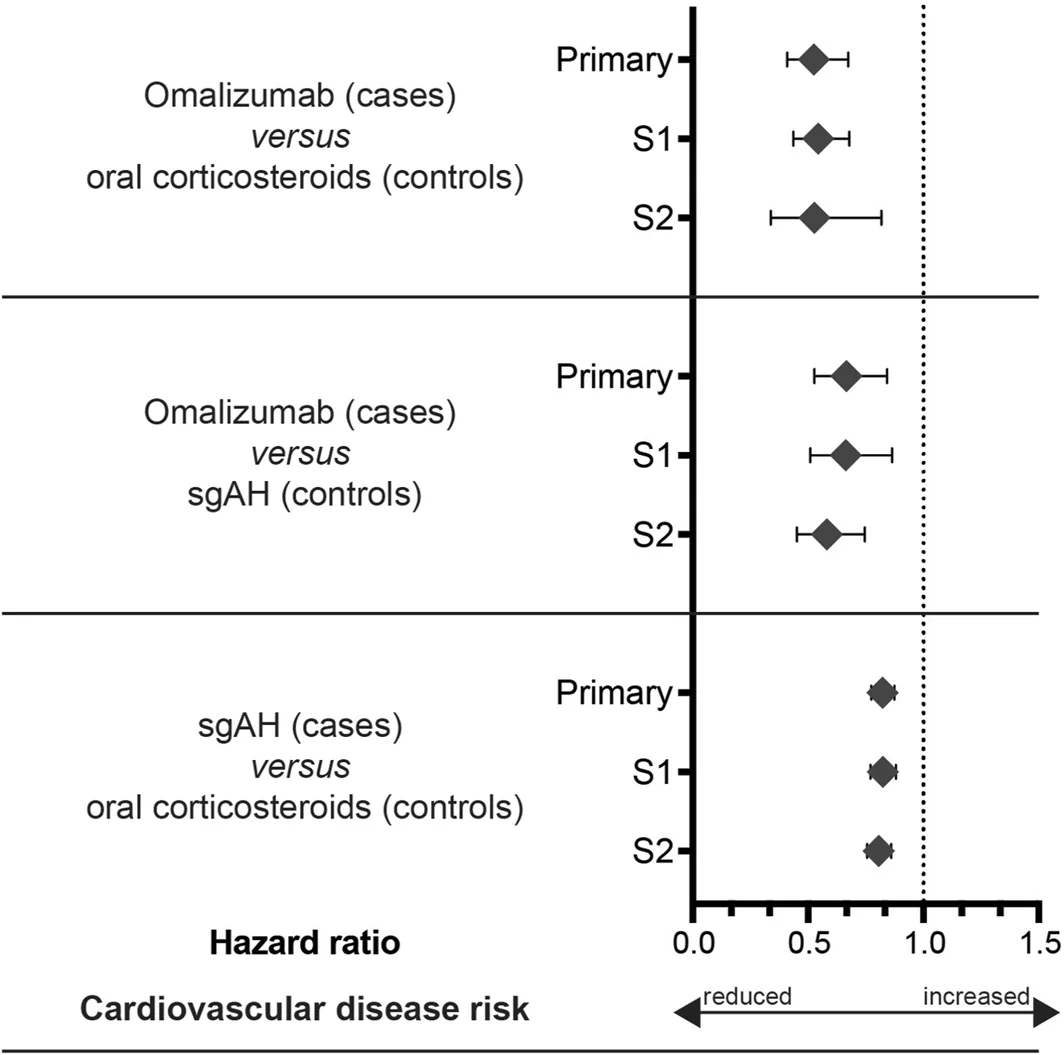
Risk of any cardiovascular disease in patients with chronic spontaneous urticaria (CSU) treated with omalizumab, second‐generation antihistamines, and oral corticosteroids: primary and sensitivity analyses. Adjusted hazard ratios (aHRs) and 95% confidence intervals for the primary analysis (1 day–3 years after index), S1: 3 months to 3 years, and S2: mandatory follow‐up between 1 and 3 years after index. *p* < 0.05 for all depicted comparisons. sgAH, second generation antihistamines.

The secondary outcomes suggested a similar hierarchy in effect estimates (OMA < sgAH < oCS), especially for MACE and VTE, though several comparisons did not reach statistical significance (likely due to low event counts). Conduction disorders and valve disorders showed inconsistent precision across windows, making any drug‐specific inference premature; these are therefore best regarded as hypothesis‐generating only (Supporting Information S1: Table 3–5).

These findings align with current understanding of CSU as a chronic inflammatory disease with potential systemic sequelae [1] and are compatible with, but do not demonstrate, a contribution of more effective symptom control to CVD risk mitigation. OMA is both guideline‐recommended and generally well tolerated [5], and its consistently favorable cardiovascular profile across analyses is reassuring from a safety perspective. However, this interpretation warrants caution: treatment allocation was not random, medication selection may capture disease severity, the reciprocal exclusion of oral corticosteroids after index may have selected omalizumab‐treated patients with more stable disease, patients receiving omalizumab may undergo more intensive medical surveillance and differ in socioeconomic status, which influences access to omalizumab in the US and was only crudely captured. Further, spontaneous remission may have biased treatment assignment and persistence, and residual confounding cannot be excluded. As disease control was not captured in this dataset, whether the observed lower risk reflects drug‐specific effects, better CSU control, or neither cannot be distinguished from these data.

In conclusion, in a large real‐world cohort of CSU patients, CVD risk differed by treatment choice. OMA was associated with consistently lower CVD risk compared with oCS or sgAH. These results support the relevance of CVD risk assessment in CSU management, but should not be interpreted as evidence of a cardioprotective effect of OMA. Future prospective studies and replication in settings with universal healthcare access should clarify whether the lower risk reflects treatment effects, disease characteristics, or residual confounding.

## Author Contributions

**Ralf J. Ludwig:** conceptualization, investigation, funding acquisition, writing – original draft, methodology, writing – review and editing, project administration, data curation, resources. **Diamant Thaci:** conceptualization, investigation, validation, supervision. **Philip Curman:** conceptualization, investigation, writing – original draft, methodology, validation, writing – review and editing, formal analysis, project administration.

## Funding

This work was supported by Novartis, the Cluster of Excellence Precision Medicine in Chronic Inflammation (EXC 2167), the Collaborative Research Center Pathomechanisms of Antibody‐mediated Autoimmunity (SFB 1626), Individual Research Grant LU 877/25‐1, by the Deutsche Forschungsgemeinschaft, and the Schleswig‐Holstein Excellence‐Chair Program from the ^. PC was supported by Region Stockholm, Karolinska Institutet, Hudfonden, the Tore Nilson Foundation, and the Swedish Society for Dermatology and Venereology.

## Ethics Statement

Approved by the Swedish Ethical Review Authority (diary number 2025‐03805‐02).

## Conflicts of Interest

D.T. has received honoraria or fees for serving on advisory boards, as a speaker and as a consultant, from AbbVie, Amgen, Almirall, Boehringer Ingelheim, Bristol Meyers Squibb, Galderma, Hexal, Janssen‐Cilag, LEO Pharma, Lilly, L’Oréal, New Bridge, Novartis, Pfizer, Regeneron, Sanofi, Sun Pharmaceuticals, UCB and Vichy. R.J.L. and P.C. has received travel grants from TriNetX. R.J.L has received funding from Novartis to support this study.

## Supporting information


Supporting Information S1

## Data Availability

The data that support the findings of this study are available from the corresponding author upon reasonable request.
