# Supplementary material for: Treatment Choice and Cardiovascular Risk in Chronic Spontaneous Urticaria
Source: Clin Transl Allergy. 2026 Oct 21;16(10):e70219. doi: 10.1002/clt2.70219 (PMC13685820; doi:10.1002/clt2.70219)
Supplement: Supplementary file 1 [file CLT2-16-e70219-s001.docx]

**Supplementary figure 1.** Flowchart of the study design.

**Supplementary table 1.** Detailed baseline characteristics for all cohorts and comparisons. Variables are reported before and after matching for the primary study cohorts. Definitions for characteristics are in International classification of diseases, 10th edition, clinical modification (ICD-10-CM) codes for comorbidities and RxNorm for drugs.

*Abbreviations: oCS: oral corticosteroids; OMA: omalizumab; sgAH: second-generation antihistamines.*

| **Omalizumab vs oral corticosteroids** | | | | | | | |
| --- | --- | --- | --- | --- | --- | --- | --- |
| **Characteristic** | **Definition** | **Before matching** | | | **After matching** | | |
|  |  | **OMA** | **oCS** | **Std. diff.** | **OMA** | **oCS** | **Std. diff.** |
| n | – | 3,303 | 44,61 | – | 3,302 | 3,302 | – |
| Age at index (years, SD) | – | 43.2 ± 16.0 | 44.1 ± 15.9 | 0.051 | 43.2 ± 16.0 | 43.6 ± 16.0 | 0.021 |
| Female (%) | – | 76.5 | 76.9 | 0.009 | 76.6 | 77.4 | 0.021 |
| Asian (%) | – | 4.1 | 8.4 | 0.175 | 4.1 | 4.2 | 0.003 |
| Black (%) | – | 9.4 | 16.1 | 0.201 | 9.4 | 8.5 | 0.034 |
| Hispanic or Latino (%) | – | 7.5 | 9.6 | 0.075 | 7.5 | 6.6 | 0.035 |
| White (%) | – | 73.3 | 63.9 | 0.203 | 73.3 | 75.1 | 0.042 |
| Nicotine dependence (%) | F17 | 4.8 | 12.1 | 0.264 | 4.8 | 4.1 | 0.035 |
| Overweight & obesity (%) | E66 | 13.8 | 21.0 | 0.189 | 13.8 | 12.9 | 0.028 |
| Diabetes mellitus (%) | E08–E13 | 7.1 | 9.7 | 0.095 | 7.0 | 6.0 | 0.042 |
| Hyperlipidemia (%) | E78 | 16.1 | 27.2 | 0.273 | 16.1 | 15.1 | 0.027 |
| Family history circulatory disease (%) | Z82.4 | 2.5 | 3.9 | 0.083 | 2.5 | 2.2 | 0.018 |
| Chronic kidney disease (%) | N18 | 1.1 | 2.5 | 0.106 | 1.1 | 1.2 | 0.008 |
| Chronic lower respiratory disease (%) | J40–J4A | 25.5 | 25.9 | 0.009 | 25.5 | 26.8 | 0.030 |
| Socioeconomic/psychosocial hazards (%) | Z55–Z65 | 1.8 | 3.4 | 0.102 | 1.8 | 1.5 | 0.019 |
| Doxepin (%) | 3638 | 5.6 | 2.0 | 0.187 | 5.5 | 4.4 | 0.053 |
| Nizatidine (%) | 42319 | 0.3 | 0.1 | 0.045 | 0.3 | 0.3 | <0.001 |
| Famotidine (%) | 4278 | 27.6 | 21.6 | 0.140 | 27.6 | 26.8 | 0.020 |
| Cimetidine (%) | 2541 | 0.9 | 0.7 | 0.019 | 0.9 | 0.7 | 0.024 |
| Montelukast (%) | 88249 | 32.3 | 12.3 | 0.496 | 32.3 | 31.3 | 0.021 |
|  | | | | | | | |
| **Omalizumab vs second-generation antihistamines** | | | | | | | |
| **Characteristic** | **Definition** | **Before matching** | | | **After matching** | | |
|  |  | **OMA** | **sgAH** | **Std. diff.** | **OMA** | **sgAH** | **Std. diff.** |
| n | – | 3,303 | 33,1 | – | 3,293 | 3,293 | – |
| Age at index (years, SD) | – | 43.2 ± 16.0 | 42.7 ± 15.9 | 0.035 | 43.2 ± 16.0 | 44.0 ± 16.3 | 0.046 |
| Female (%) | – | 76.5 | 77.1 | 0.013 | 76.5 | 77.5 | 0.024 |
| Asian (%) | – | 4.1 | 10.3 | 0.239 | 4.2 | 3.8 | 0.020 |
| Black (%) | – | 9.4 | 18.9 | 0.273 | 9.5 | 8.7 | 0.027 |
| Hispanic or Latino (%) | – | 7.5 | 9.5 | 0.070 | 7.5 | 7.4 | 0.006 |
| White (%) | – | 73.3 | 54.9 | 0.390 | 73.2 | 74.3 | 0.026 |
| Nicotine dependence (%) | F17 | 4.8 | 8.2 | 0.138 | 4.8 | 3.9 | 0.045 |
| Overweight & obesity (%) | E66 | 13.8 | 18.7 | 0.131 | 13.9 | 14.0 | 0.004 |
| Diabetes mellitus (%) | E08–E13 | 7.1 | 9.2 | 0.078 | 7.1 | 6.3 | 0.032 |
| Hyperlipidemia (%) | E78 | 16.1 | 23.5 | 0.187 | 16.1 | 16.7 | 0.015 |
| Family history circulatory disease (%) | Z82.4 | 2.5 | 3.2 | 0.045 | 2.5 | 2.1 | 0.022 |
| Chronic kidney disease (%) | N18 | 1.1 | 2.0 | 0.074 | 1.1 | 1.5 | 0.030 |
| Chronic lower respiratory disease (%) | J40–J4A | 25.5 | 20.8 | 0.111 | 25.4 | 27.6 | 0.049 |
| Socioeconomic/psychosocial hazards (%) | Z55–Z65 | 1.8 | 3.4 | 0.106 | 1.8 | 1.5 | 0.019 |
| Doxepin (%) | 3638 | 5.6 | 1.6 | 0.213 | 5.3 | 5.0 | 0.014 |
| Nizatidine (%) | 42319 | 0.3 | 0.1 | 0.054 | 0.3 | 0.3 | <0.001 |
| Famotidine (%) | 4278 | 27.6 | 16.0 | 0.285 | 27.5 | 27.1 | 0.007 |
| Cimetidine (%) | 2541 | 0.9 | 0.5 | 0.048 | 0.8 | 0.5 | 0.033 |
| Montelukast (%) | 88249 | 32.3 | 9.6 | 0.580 | 32.1 | 31.3 | 0.017 |
|  | | | | | | | |
| **Second-generation antihistamines vs oral corticosteroids** | | | | | | | |
| **Characteristic** | **Definition** | **Before matching** | | | **After matching** | | |
|  |  | **sgAH** | **oCS** | **Std. diff.** | **sgAH** | **oCS** | **Std. diff.** |
| n | – | 33,1 | 44,61 | – | 31,602 | 31,6 | – |
| Age at index (years, SD) | – | 42.7 ± 15.9 | 44.1 ± 15.9 | 0.087 | 42.9 ± 15.9 | 42.6 ± 15.7 | 0.014 |
| Female (%) | – | 77.1 | 76.9 | 0.004 | 77.1 | 77.1 | <0.001 |
| Asian (%) | – | 10.3 | 8.4 | 0.067 | 10.4 | 10.0 | 0.015 |
| Black (%) | – | 18.9 | 16.1 | 0.073 | 19.1 | 18.3 | 0.020 |
| Hispanic or Latino (%) | – | 9.5 | 9.6 | 0.005 | 9.6 | 9.6 | 0.002 |
| White (%) | – | 54.9 | 63.9 | 0.184 | 56.4 | 57.5 | 0.022 |
| Nicotine dependence (%) | F17 | 8.2 | 12.1 | 0.129 | 8.4 | 8.3 | 0.003 |
| Overweight & obesity (%) | E66 | 18.7 | 21.0 | 0.057 | 18.9 | 18.3 | 0.015 |
| Diabetes mellitus (%) | E08–E13 | 9.2 | 9.7 | 0.017 | 9.3 | 8.8 | 0.017 |
| Hyperlipidemia (%) | E78 | 23.5 | 27.2 | 0.086 | 24.0 | 23.0 | 0.023 |
| Family history circulatory disease (%) | Z82.4 | 3.2 | 3.9 | 0.038 | 3.3 | 3.0 | 0.016 |
| Chronic kidney disease (%) | N18 | 2.0 | 2.5 | 0.034 | 2.1 | 1.8 | 0.023 |
| Chronic lower respiratory disease (%) | J40–J4A | 20.8 | 25.9 | 0.120 | 21.4 | 21.1 | 0.005 |
| Socioeconomic/psychosocial hazards (%) | Z55–Z65 | 3.4 | 3.4 | 0.004 | 3.3 | 3.0 | 0.016 |
| Doxepin (%) | 3638 | 1.6 | 2.0 | 0.029 | 1.7 | 1.6 | 0.005 |
| Nizatidine (%) | 42319 | 0.1 | 0.1 | 0.010 | 0.1 | 0.1 | 0.002 |
| Famotidine (%) | 4278 | 16.0 | 21.6 | 0.144 | 16.4 | 16.0 | 0.010 |
| Cimetidine (%) | 2541 | 0.5 | 0.7 | 0.029 | 0.5 | 0.5 | <0.001 |
| Montelukast (%) | 88249 | 9.6 | 12.3 | 0.085 | 9.8 | 9.6 | 0.007 |

**Supplementary table 2.** Detailed definitions for all study outcomes. Groups of international classification of diseases, 10^th^ edition, clinical modification (ICD-10-CM) codes and their corresponding descriptions for all study outcomes.

| **Outcome** | **Definition** |
| --- | --- |
| **Any CVD** | Acute myocardial infarction I21; or |
|  | Subsequent ST elevation (STEMI) and non-ST elevation (NSTEMI) myocardial infarction I22; or |
|  | Cardiac arrest I46; or |
|  | Nontraumatic intracerebral hemorrhage I61; or |
|  | Other and unspecified nontraumatic intracranial hemorrhage I62; or |
|  | Cerebral infarction I63; or |
|  | Cardiogenic shock R57.0; or |
|  | Pulmonary embolism I26; or |
|  | Acute embolism and thrombosis of deep veins of lower extremity I82.4; or |
|  | Rheumatic fever with heart involvement I01; or |
|  | Rheumatic chorea I02; or |
|  | Rheumatic mitral valve diseases I05; or |
|  | Rheumatic aortic valve diseases I06; or |
|  | Rheumatic tricuspid valve diseases I07; or |
|  | Other rheumatic heart diseases I09; or |
|  | Atrioventricular and left bundle-branch block I44; or |
|  | Other conduction disorders I45; or |
|  | Paroxysmal tachycardia I47; or |
|  | Atrial fibrillation and flutter I48; or |
|  | Other cardiac arrhythmias I49; or |
|  | Presence of cardiac pacemaker Z95.0; or |
|  | Pericarditis in diseases classified elsewhere I32; or |
|  | Endocarditis, valve unspecified I38; or |
|  | Acute myocarditis I40; or |
|  | Myocarditis in diseases classified elsewhere I41; or |
|  | Cardiomyopathy in diseases classified elsewhere I43; or |
|  | Myocarditis, unspecified I51.4; or |
|  | Influenza due to other identified influenza virus with myocarditis J10.82; or |
|  | Viral carditis B33.2; or |
|  | Nonrheumatic mitral valve disorders I34; or |
|  | Nonrheumatic aortic valve disorders I35; or |
|  | Nonrheumatic tricuspid valve disorders I36; or |
|  | Presence of prosthetic heart valve Z95.2; or |
|  | Presence of xenogenic heart valve Z95.3; or |
|  | Presence of other heart-valve replacement Z95.4. |
| **Major adverse cardiovascular events** | Acute myocardial infarction I21; or |
|  | Subsequent ST elevation (STEMI) and non-ST elevation (NSTEMI) myocardial infarction I22; or |
|  | Cardiac arrest I46; or |
|  | Heart failure I50; or |
|  | Nontraumatic intracerebral hemorrhage I61; or |
|  | Cerebral infarction I63; or |
|  | Cardiogenic shock R57.0. |
| **Venous thromboembolism** | Pulmonary embolism I26; or |
|  | Acute embolism and thrombosis of deep veins of lower extremity I82.4. |
| **Rheumatic heart disease** | Rheumatic fever with heart involvement I01; or |
|  | Rheumatic chorea I02; or |
|  | Rheumatic mitral valve diseases I05; or |
|  | Rheumatic aortic valve diseases I06; or |
|  | Rheumatic tricuspid valve diseases I07; or |
|  | Other rheumatic heart diseases I09. |
| **Conduction disorders** | Atrioventricular and left bundle-branch block I44; or |
|  | Other conduction disorders I45. |
| **Valve disorders** | Nonrheumatic mitral valve disorders I34; or |
|  | Nonrheumatic aortic valve disorders I35; or |
|  | Nonrheumatic tricuspid valve disorders I36; or |
|  | Nonrheumatic pulmonary valve disorders I37; or |
|  | Presence of prosthetic heart valve Z95.2; or |
|  | Presence of xenogenic heart valve Z95.3; or |
|  | Presence of other heart-valve replacement Z95.4. |
| **Carditis** | Pericarditis in diseases classified elsewhere I32; or |
|  | Endocarditis, valve unspecified I38; or |
|  | Acute myocarditis I40; or |
|  | Myocarditis in diseases classified elsewhere I41. |
| **Hernia (negative control** | Hernia K40-K46. |

**Supplementary table 3.** Results for the main outcome and all secondary outcomes for the comparison between omalizumab and oral corticosteroids. Adjusted hazard ratios (aHRs) and 95% confidence intervals for the primary analysis (1 day to 3 years after index), S1: 3 months to 3 years, and S2: mandatory follow-up between 1–3 years after index.

*Abbreviations: CI: confidence interval.*

| **Outcome** | **Omalizumab** | | | **Oral corticosteroids** | | | **Hazard ratio (95% CI)** | **P-value** |
| --- | --- | --- | --- | --- | --- | --- | --- | --- |
|  | **N total** | **N outcomes** | **Risk (%)** | **N total** | **N outcomes** | **Risk (%)** |  |  |
|  | | | | | | | | |
| **Primary: 1 day to 3 years** | | | | | | | | |
| Any cardiovascular disease | 3,185 | 113 | 3.55 | 3,202 | 258 | 8.06 | 0.544 (0.436–0.679) | <0.001 |
| Major adverse cardiovascular events | 3,283 | 28 | 0.85 | 3,266 | 69 | 2.11 | 0.527 (0.339–0.818) | 0.004 |
| Venous thromboembolism | 3,284 | 15 | 0.46 | 3,291 | 36 | 1.09 | 0.527 (0.288–0.964) | 0.034 |
| Rheumatic heart disease | 3,300 | 12 | 0.36 | 3,3 | 21 | 0.64 | 0.711 (0.349–1.448) | 0.345 |
| Conduction disorders | 3,282 | 14 | 0.43 | 3,287 | 45 | 1.37 | 0.386 (0.212–0.705) | 0.001 |
| Valve disorders | 3,276 | 31 | 0.95 | 3,29 | 60 | 1.82 | 0.673 (0.436–1.039) | 0.072 |
| Hernia (negative control) | 3,174 | 57 | 1.80 | 3,12 | 86 | 2.76 | 0.820 (0.586–1.147) | 0.246 |
|  | | | | | | | | |
| **S1: 3 months to 3 years** | | | | | | | | |
| Any cardiovascular disease | 3,159 | 87 | 2.75 | 3,159 | 215 | 6.81 | 0.525 (0.409–0.674) | <0.001 |
| Major adverse cardiovascular events | 3,276 | 21 | 0.64 | 3,262 | 65 | 1.99 | 0.432 (0.264–0.708) | 0.001 |
| Venous thromboembolism | 3,282 | 13 | 0.40 | 3,283 | 28 | 0.85 | 0.619 (0.320–1.196) | 0.149 |
| Rheumatic heart disease | 3,298 | 10 | 0.30 | 3,296 | 17 | 0.52 | 0.764 (0.349–1.671) | 0.499 |
| Conduction disorders | ***Too few outcomes*** | | | | | | | |
| Valve disorders | 3,271 | 26 | 0.79 | 3,284 | 54 | 1.64 | 0.648 (0.406–1.035) | 0.067 |
|  | | | | | | | | |
| **S2: 1 day to 3 years, mandatory visit 1–3 years after index** | | | | | | | | |
| Any cardiovascular disease | 2,256 | 95 | 4.21 | 2,257 | 208 | 9.22 | 0.515 (0.404–0.657) | <0.001 |
| Major adverse cardiovascular events | 2,337 | 24 | 1.03 | 2,323 | 57 | 2.45 | 0.484 (0.300–0.781) | 0.002 |
| Venous thromboembolism | 2,336 | 14 | 0.60 | 2,338 | 32 | 1.37 | 0.502 (0.268–0.941) | 0.028 |
| Rheumatic heart disease | 2,349 | 10 | 0.43 | 2,349 | 24 | 1.02 | 0.477 (0.228–0.998) | 0.044 |
| Conduction disorders | ***Too few outcomes*** | | | | | | | |
| Valve disorders | 2,333 | 26 | 1.11 | 2,334 | 57 | 2.44 | 0.533 (0.335–0.847) | 0.007 |

**Supplementary table 4.** Results for the main outcome and all secondary outcomes for the comparison between omalizumab and second-generation antihistamines. Adjusted hazard ratios (aHRs) and 95% confidence intervals for the primary analysis (1 day to 3 years after index), S1: 3 months to 3 years, and S2: mandatory follow-up between 1–3 years after index.

*Abbreviations: CI: confidence interval.*

| **Outcome** | **Omalizumab** | | | **Second-generation antihistamines** | | | **Hazard ratio (95% CI)** | **P-value** |
| --- | --- | --- | --- | --- | --- | --- | --- | --- |
|  | **N total** | **N outcomes** | **Risk (%)** | **N total** | **N outcomes** | **Risk (%)** |  |  |
|  | | | | | | | | |
| **Primary: 1 day to 3 years** | | | | | | | | |
| Any cardiovascular disease | 3,177 | 112 | 3.52 | 3,176 | 192 | 6.05 | 0.667 (0.528–0.842) | 0.001 |
| Major adverse cardiovascular events | 3,274 | 26 | 0.79 | 3,259 | 50 | 1.53 | 0.600 (0.374–0.965) | 0.033 |
| Venous thromboembolism | 3,275 | 14 | 0.43 | 3,279 | 18 | 0.55 | 0.888 (0.441–1.786) | 0.738 |
| Rheumatic heart disease | 3,291 | 12 | 0.36 | 3,287 | 14 | 0.43 | 1.012 (0.468–2.190) | 0.976 |
| Conduction disorders | 3,274 | 14 | 0.43 | 3,27 | 31 | 0.95 | 0.516 (0.275–0.971) | 0.037 |
| Valve disorders | 3,267 | 31 | 0.95 | 3,263 | 46 | 1.41 | 0.785 (0.497–1.238) | 0.297 |
| Hernia (negative control) | 3,166 | 55 | 1.74 | 3,14 | 78 | 2.48 | 0.810 (0.573–1.144) | 0.230 |
|  | | | | | | | | |
| **S1: 3 months to 3 years** | | | | | | | | |
| Any cardiovascular disease | 3,151 | 86 | 2.73 | 3,137 | 153 | 4.88 | 0.664 (0.510–0.865) | 0.002 |
| Major adverse cardiovascular events | 3,267 | 19 | 0.58 | 3,251 | 42 | 1.29 | 0.540 (0.314–0.928) | 0.024 |
| Venous thromboembolism | 3,273 | 12 | 0.37 | 3,275 | 14 | 0.43 | 1.004 (0.464–2.171) | 0.992 |
| Rheumatic heart disease | 3,289 | 10 | 0.30 | 3,286 | 13 | 0.40 | 0.926 (0.406–2.112) | 0.854 |
| Conduction disorders | ***Too few outcomes*** | | | | | | | |
| Valve disorders | 3,262 | 26 | 0.80 | 3,254 | 37 | 1.14 | 0.845 (0.512–1.396) | 0.511 |
|  | | | | | | | | |
| **S2: 1 day to 3 years, mandatory visit 1–3 years after index** | | | | | | | | |
| Any cardiovascular disease | 2,244 | 94 | 4.19 | 2,24 | 178 | 7.95 | 0.581 (0.452–0.746) | <0.001 |
| Major adverse cardiovascular events | 2,325 | 23 | 0.99 | 2,312 | 39 | 1.69 | 0.647 (0.386–1.084) | 0.095 |
| Venous thromboembolism | 2,324 | 14 | 0.60 | 2,322 | 14 | 0.60 | 1.130 (0.538–2.371) | 0.747 |
| Rheumatic heart disease | 2,337 | 10 | 0.43 | 2,335 | 18 | 0.77 | 0.621 (0.287–1.347) | 0.223 |
| Conduction disorders | ***Too few outcomes*** | | | | | | | |
| Valve disorders | 2,321 | 26 | 1.12 | 2,325 | 51 | 2.19 | 0.578 (0.360–0.927) | 0.021 |

**Supplementary table 5.** Results for the main outcome and all secondary outcomes for the comparison between second-generation antihistamines and oral corticosteroids. Adjusted hazard ratios (aHRs) and 95% confidence intervals for the primary analysis (1 day to 3 years after index), S1: 3 months to 3 years, and S2: mandatory follow-up between 1–3 years after index.

*Abbreviations: CI: confidence interval.*

| **Outcome** | **Second-generation antihistamines** | | | **Oral corticosteroids** | | | **Hazard ratio (95% CI)** | **P-value** |
| --- | --- | --- | --- | --- | --- | --- | --- | --- |
|  | **N total** | **N outcomes** | **Risk (%)** | **N total** | **N outcomes** | **Risk (%)** |  |  |
|  | | | | | | | | |
| **Primary: 1 day to 3 years** | | | | | | | | |
| Any cardiovascular disease | 30,547 | 1,787 | 5.85 | 30,759 | 2,341 | 7.62 | 0.823 (0.774–0.875) | <0.001 |
| Major adverse cardiovascular events | 31,171 | 460 | 1.48 | 31,235 | 605 | 1.94 | 0.822 (0.728–0.928) | 0.002 |
| Venous thromboembolism | 31,481 | 202 | 0.64 | 31,505 | 300 | 0.95 | 0.726 (0.607–0.868) | <0.001 |
| Rheumatic heart disease | 31,547 | 105 | 0.33 | 31,556 | 144 | 0.46 | 0.786 (0.611–1.011) | 0.060 |
| Conduction disorders | 31,426 | 302 | 0.96 | 31,477 | 431 | 1.37 | 0.756 (0.653–0.876) | <0.001 |
| Valve disorders | 31,340 | 460 | 1.47 | 31,415 | 510 | 1.62 | 0.978 (0.863–1.110) | 0.277 |
| Carditis | 31,586 | 37 | 0.12 | 31,592 | 37 | 0.12 | 1.079 (0.684–1.701) | 0.529 |
| Hernia (negative control) | 30,054 | 715 | 2.38 | 30,003 | 974 | 3.25 | 0.787 (0.715–0.867) | <0.001 |
|  | | | | | | | | |
| **S1: 3 months to 3 years** | | | | | | | | |
| Any cardiovascular disease | 30,273 | 1,513 | 5.00 | 30,419 | 2,001 | 6.58 | 0.824 (0.770–0.881) | <0.001 |
| Major adverse cardiovascular events | 31,113 | 402 | 1.29 | 31,155 | 525 | 1.69 | 0.836 (0.734–0.952) | 0.007 |
| Venous thromboembolism | 31,458 | 179 | 0.57 | 31,462 | 257 | 0.82 | 0.758 (0.627–0.918) | 0.004 |
| Rheumatic heart disease | 31,537 | 95 | 0.30 | 31,529 | 117 | 0.37 | 0.885 (0.675–1.160) | 0.377 |
| Conduction disorders | 31,380 | 256 | 0.82 | 31,419 | 373 | 1.19 | 0.749 (0.638–0.878) | <0.001 |
| Valve disorders | 31,268 | 388 | 1.24 | 31,354 | 449 | 1.43 | 0.948 (0.827–1.086) | 0.439 |
| Carditis | 31,581 | 32 | 0.10 | 31,585 | 30 | 0.10 | 1.165 (0.708–1.918) | 0.547 |
|  | | | | | | | | |
| **S2: 1 day to 3 years, mandatory visit 1–3 years after index** | | | | | | | | |
| Any cardiovascular disease | 24,742 | 1,672 | 6.76 | 24,892 | 2,139 | 8.59 | 0.806 (0.756–0.860) | <0.001 |
| Major adverse cardiovascular events | 25,301 | 433 | 1.71 | 25,348 | 597 | 2.36 | 0.752 (0.665–0.851) | <0.001 |
| Venous thromboembolism | 25,575 | 192 | 0.75 | 25,591 | 269 | 1.05 | 0.738 (0.613–0.888) | 0.001 |
| Rheumatic heart disease | 25,633 | 96 | 0.37 | 25,64 | 130 | 0.51 | 0.762 (0.586–0.992) | 0.043 |
| Conduction disorders | 25,530 | 286 | 1.12 | 25,554 | 385 | 1.51 | 0.769 (0.660–0.897) | 0.001 |
| Valve disorders | 25,444 | 433 | 1.70 | 25,51 | 480 | 1.88 | 0.938 (0.824–1.068) | 0.333 |
| Carditis | 25,669 | 35 | 0.14 | 25,672 | 41 | 0.16 | 0.891 (0.568–1.399) | 0.616 |
